# Comparing Complementary and Alternative Medicine Use with or without Including Prayer as a Modality in a Local and Diverse United States Jurisdiction

**DOI:** 10.3389/fpubh.2017.00056

**Published:** 2017-03-21

**Authors:** Brenda Robles, Dawn M. Upchurch, Tony Kuo

**Affiliations:** ^1^Division of Chronic Disease and Injury Prevention, Los Angeles County Department of Public Health, Los Angeles, CA, USA; ^2^Department of Community Health Sciences, UCLA Fielding School of Public Health, Los Angeles, CA, USA; ^3^Department of Epidemiology, UCLA Fielding School of Public Health, Los Angeles, CA, USA; ^4^Department of Family Medicine, David Geffen School of Medicine at UCLA, Los Angeles, CA, USA

**Keywords:** complementary and alternative medicine, prayer, public health, surveillance, benchmarking

## Abstract

**Objectives:**

Few studies to date have examined the utilization of complementary and alternative medicine (CAM) in a local, ethnically diverse population in the United States (U.S.). Fewer have addressed the differences in their use based on inclusion or exclusion of prayer as a modality. Variable definitions of CAM are known to affect public health surveillance (i.e., continuous, systematic data collection, analysis, and interpretation) or benchmarking (i.e., identifying and comparing key indicators of health to inform community planning) related to this non-mainstream collection of health and wellness therapies. The present study sought to better understand how including or excluding prayer could affect reporting of CAM use among residents of a large, urban U.S. jurisdiction.

**Design:**

Using population-weighted data from a cross-sectional Internet panel survey collected as part of a larger countywide population health survey, the study compared use of CAM based on whether prayer or no prayer was included in its definition. Patterns of CAM use by socio-demographic characteristics were described for the two operationalized definitions. Multivariable binomial regression analyses were performed to control for gender, age, race/ethnicity, education, employment, income, and health insurance status. One of the analyses explored the associations between CAM use and racial/ethnic characteristics in the study sample.

**Setting:**

Los Angeles County, California.

**Subjects:**

A socio-demographically diverse sample of Los Angeles County residents.

**Outcome measures:**

CAM use (with prayer) and CAM use (excluding prayer).

**Results:**

Blacks were among the highest users of CAM when compared to Whites, especially when prayer was included as a CAM modality. Regardless of prayer inclusion, being a woman predicted higher use of CAM.

**Conclusions:**

How CAM is defined matters in gauging the utilization of this non-mainstream collection of therapies. Given that surveillance and/or benchmarking data are often used to inform resource allocation and planning decisions, results from the present study suggest that when prayer is included as part of the CAM definition, utilization/volume estimates of its use increased correspondingly, especially among non-White residents of the region.

## Introduction

Complementary and alternative medicine (CAM) encompasses a wide range of practices, procedures, and products that are used together with or in place of conventional medicine (1, 2). In the past two decades, there has been considerable amount of attention paid to describing CAM utilization among adults in the United States (U.S.) (1–8). For example, about one-third of Americans used some form of CAM in 2007; this use corresponded to over $33.9 billion in out-of-pocket costs (9).

Although regional variation in CAM use exists (e.g., prevalence is highest in the Western states) (1, 6, 10), this variation is not entirely well-characterized in the literature. While such variation in use could be attributed to differences in cultural norms and attitudes toward these modalities (11), an alternative explanation could be that CAM has been defined differently across studies.

Presently, there is no agreed upon definition of CAM or for its pattern of use. Increasingly, even the term itself, “complementary and alternative medicine,” has been replaced by newer descriptors such as “complementary health approaches,” “integrative medicine,” or “integrative health” (12, 13). This inconsistent operationalization of CAM has and will continue to alter the core activities of public health practice. For example, surveillance and benchmarking of key health indicators or the volume of services utilization could fluctuate depending on how CAM is defined or measured. This, in turn, affects local planning of health and human services.

Surveillance is defined by the public health profession as “the continuous, systematic collection, analysis, and interpretation of health-related data needed for planning, implementation, and evaluation of public health practices” (14). Benchmarking, which complements surveillance, refers to “the comparison of indicators in a time-limited approach”; this method is often used to guide decisions about where to invest public health resources (15). Both surveillance and benchmarking possess utility as a continuous quality improvement strategy which can be used to drive health and health-care decision-making (15, 16). In the County of Los Angeles, for example, the Los Angeles County Department of Public Health (DPH) utilizes both surveillance and benchmarking data to guide decisions about funding for health and human services, community programming, and consumer protection messaging that target priority populations.

The emerging dialog on whether to incorporate “prayer” into the definition of CAM is timely and an appropriate question to answer, given the growing movement toward value-based care in the U.S. (17). Empirical evidence in the literature suggests that prayer is associated with race, ethnicity, and socioeconomic status (18–22). Specifically, ethnic-minorities and individuals of lower socioeconomic status are often the groups that pray most often and are the most likely to combine treatments that are more non-traditional or outside mainstream medicine, typically without any disclosure to their physicians (19–22). Because of this nexus between CAM and mainstream medicine, excluding prayer from the operational definition of CAM could lead to unintended consequences—e.g., health behaviors based on faith and spiritual values that are not disclosed to providers may impact patient adherence to recommended medical treatments. Since the actual volume or impact of CAM utilization or substitute care in communities of color and/or other at-risk populations could be large, a less than robust surveillance and benchmarking of these patterns of use (and their potential consequences) may inadvertently lead to marginalization of the needs of these communities, thereby creating disparities. When past national estimates did not include prayer in their operational definition, researchers found that CAM use was more common among Whites as compared to non-Hispanic Blacks (1, 2, 5, 6, 8). In contrast, when researchers considered prayer as a CAM modality in the operational definition, the results showed an opposite pattern of use (8, 23–27).

The present study sought to contribute to this gap in public health practice and to the dialogs about CAM integration by comparing CAM use across a socio-demographically diverse sample of Los Angeles County residents, based on whether prayer or no prayer (broadly defined) was included as part of the operational definition. The diversity in Los Angeles County makes this jurisdiction a prime study location for examining this subject matter. Almost three-fourths (73.4%) of its 10 million residents belong to a minority race/ethnic group: Blacks or African-Americans, Hispanics, Asians, Pacific Islanders, American Indians, or Alaskan Natives (28). Results from this study will inform health and human services delivery and planning of wellness and community resource centers in Los Angeles County.

## Methods

### Study Design and Sample

The present study utilized data from the Los Angeles County DPH Clinical Services Survey, a cross-sectional Internet panel survey conducted during June–July, 2014. This survey was commissioned by DPH to a national firm specializing in online panel surveys. Los Angeles County adults aged 18 years and older were recruited from a panel of subscribers, which were selected by a sub-contracted online panel provider. This panel provider generally invites prospective respondents with pre-existing relationships to globally recognized and business-focused companies such as Delta Airlines and Macy’s to join the survey panel and be screened for eligibility into various surveys. In addition to the age criterion, the respondents of the DPH survey had to meet quota targets created for socio-demographics that were aligned with the 2012 American Community Survey (ACS) and the 2011 Los Angeles County Health Survey (LACHS). The quota targets allowed the vendor to recruit a sample that, as closely as possible, represented the socio-demographic distributions of the region’s service planning areas.

To account for differential sampling rates (e.g., panel under-coverage) and differential non-responses (e.g., harder-to-reach subgroups such as younger minority men with lower incomes), and to adjust for other parameters such as marital status, education, parental status, poverty level, and insurance, the survey firm (vendor) created sample weights and applied them to the survey data. Although the 2012 ACS census data were the primary source or mainstay for the target quotas and weighting schemes, the vendor used the 2011 LACHS data to fill in the gaps for demographic information missing from census; specifically, the vendor consulted with the LACHS as a way to confirm census information and to ensure that viable ranges for demographic groups were captured when they did not exactly match up to the ACS.

The survey was administered in English or Spanish through the firm’s web-based platform. With the exception of “other” response options, all questions were closed ended. When feasible, questions were taken or adapted from other national or local population-based health surveys; they were pretested before fielding. All survey protocols and materials were reviewed and approved by the DPH Institutional Review Board prior to field implementation. Administration of this survey has been described elsewhere (29).

A total of 1,044 adults completed the full online survey. The adjusted response rate was approximately 32% (1,044/3,252). When weights were applied to the estimates, the demographic composition of the sample was aligned with population estimates reported by the Census Bureau for Los Angeles County (28). The final analysis sample size was 1,044 adults who completed the full online survey and the adjusted response rate was approximately 32% (1,044/2,252).

### Measures

#### CAM Use

Complementary and alternative medicine use was measured in the DPH Clinical Services Survey using two questions adapted from previous national surveys focused on measuring CAM. The first question was adapted from the National Health Interview Survey (30, 31). Respondents were asked, “In the past 12 months, which of the following have you used or done?” Response options included: (1) acupuncture; (2) biofeedback; (3) chiropractic manipulation; (4) cleanse; (5) commercial diet; (6) energy healing/reiki; (7) folk remedies; (8) homeopathy; (9) herbs/botanicals; (10) hypnosis; (11) imagery; (12) lifestyle, diet; (13) massage therapy; (14) meditation; (15) megavitamins/dietary supplements; (16) relaxation techniques; (17) self-help groups; (18) self-prayer (or prayer); (19) spiritual healing by other; (20) yoga; and (21) other (open-ended). For each reported CAM modality, responses were stratified as “yes” if a respondent checked off the response option or “no” if they did not. The second question was adapted from the Consumer Assessment of Healthcare Providers and System survey. Respondents were asked, “When was your most recent visit to a naturopath?” Response options included “in the last month,” “in the last six months,” “in the last year,” “in the last 1–2 years,” “in the last 2–5 years,” “over 5 years ago,” and “have never seen this type of medical professional.” Responses of “in the last month,” “in the last six months,” or “in the last year” were coded as “yes” for the purpose of combining the two CAM use measures into a single measure.

In this study, CAM use was operationalized in two ways: (1) as an outcome variable classified as “All CAM” that was a sum of all “yes” responses to the two aforementioned CAM questions (i.e., including prayer/self-prayer); and (2) as an outcome variable that was classified as “All CAM (excluding prayer).” The latter was a sum of all “yes” responses to the two aforementioned CAM questions, but excluded prayer/self-prayer. Spiritual healing by others was not classified as prayer and included in both operational definitions.

#### Socio-Demographics

Gender was a dichotomous variable. Age was coded ordinally as 18–24, 25–44, 45–64, and 65+. Race and ethnicity were based on self-report and coded into five mutually exclusive categories: White, Black, Hispanic/Latino, Asian/Pacific Islander, and other (the latter included the race/ethnicity response options American Indian/Alaska Native and other). Education was coded into five categories: <high school, high school graduate, some college, technical/vocational degree, and college or postgraduate degree. Employment was coded categorically as employed full time, employed part time, unemployed, retired, and student/homemaker. Total family income was an ordinal variable (≤$24,999, $25,000–$49,000, $50,000–$74,999, $75,000–$99,999, and ≥$100,000). Finally, health insurance status was coded as uninsured, private, and public.

### Statistical Analysis

All analyses and estimates used individual-level sampling weights. Descriptive statistics and bivariate prevalence estimates of CAM use were generated for reporting in the tables. Multivariable binomial logistic regression analyses were performed to examine the associations between race and ethnicity and CAM use (including and excluding prayer), while controlling for other socio-demographic characteristics. Associations between the top 14 commonly used CAM modalities (compared by race/ethnicity) were also examined. A sub-analysis was conducted to better understand the relationship between prayer use and socio-demographic characteristics. Data pertaining to associations between the top 14 commonly used CAM modalities was cleaned and managed using the SAS version 9.3 statistical software package. All other analyses were performed using STATA version 14.0 (StataCorp LP, College Station, TX, USA).

## Results

Table 1 presents the socio-demographic characteristics of all adults interviewed and their use, according to the two operational definitions of CAM. The majority of respondents were Hispanic/Latino (43%), followed by White (31%), Asian/Pacific Islander (16%), and Black (9%). The percentages of men and women were comparable, 49% and 51%, respectively. Over half of the respondents were under age 45 (54%). About one-third of them had completed college (35%) and over a third had some college or attended technical school (37%). Half were employed full time, 10% were unemployed, and about 15% were retired. About a quarter reported family incomes between $25,000 and $49,999 (24%) and over $100,000 (24%), respectively. More than 86% reported having some kind of health insurance. Overall, the gender, race, and income characteristics of the respondents aligned closely with the Los Angeles County population estimates reported by the U.S. Census Bureau (*comparison data table not shown*). Only age was more skewed toward the 25–64 age range.

**Table 1:** **Socio-demographic characteristics of respondents from the 2014 internet-based Los Angeles County Clinical Services Survey and percentages of complementary and alternative medicine (CAM) use, including and excluding prayer (*n* = 1,044)**.

	Total (*n* = 1,044)		All CAM^a^ (*n* = 696)		CAM (excluding prayer)^b^ (*n* = 637)	
Characteristics	*n*	% (weighted)	% (weighted)^c^	*p*-Value^d^	% (weighted)^c^	*p*-Value^d^
**Total CAM use**			63.5		56.7	
Race/ethnicity				<0.05		–
Black	121	8.6	79.1		59.8	
Hispanic/Latino	339	43.0	65.3		59.7	
White	406	30.9	61.2		56.0	
Asian/Pacific Islander	161	16.1	56.8		49.5	
Other	17	1.6	43.9		43.9	
Gender				<0.05		<0.05
Women	636	51.3	67.7		61.7	
Men	408	48.7	59.2		51.5	
Age (years)				–		–
18–24	94	12.7	59.4		56.5	
25–44	438	40.8	67.4		62.9	
45–64	388	33.0	64.1		53.1	
65+	124	13.6	54.5		47.2	
Education				–		–
Less than high school	14	3.5	39.1		39.1	
High school graduate	143	24.6	60.5		50.2	
Some college	303	26.9	62.1		55.7	
Technical/vocational degree	114	10.2	68.9		60.9	
College graduate or postgraduate	466	34.5	67.3		62.7	
Employment				–		<0.01
Employed full time	591	50.3	65.9		60.6	
Employed part time	116	12.4	73.7		68.6	
Unemployed	93	10.3	57.4		47.3	
Retired	150	15.2	55.5		44.3	
Student/homemaker	94	11.7	58.5		51.7	
Income				–		–
<$15,000–$24,999	134	19.7	62.9		51.9	
$25,000–$49,000	236	24.1	57.9		50.7	
$50,000–$74,000	229	18.6	64.8		60.2	
$75,000–$99,000	165	13.2	61.7		57.1	
$100,000+	280	24.4	69.6		63.8	
Health insurance status				–		–
Private	746	58.1	65.6		59.4	
Public	210	28.1	58.8		51.7	
Uninsured	56	10.7	68.6		60.9	

*^a^Operationalized as all CAM modalities (representing the category “All CAM”), including acupuncture, biofeedback, chiropractic manipulation, cleanse, commercial diet, energy healing/reiki, folk remedies, homeopathy, herbs/botanicals, hypnosis, imagery, lifestyle diet, massage therapy, meditation, megavitamins/dietary supplements, relaxation techniques, self-help groups, self-prayer, spiritual healing by other, yoga, and other (open-ended)*.

*^b^Operationalized as including all CAM modalities in “All CAM” category, except for self-prayer*.

*^c^Weighted row percentages reported only for CAM users; number of cases and percentage do not add up to the total or 100% due to rounding or missing, “don’t know” or “other” responses*.

*^d^Weighted chi-squared test*.

Comparing the two operational definitions of CAM, about 64% reported use of all CAM in the past 12 months; this percentage was lower (57%) when prayer was excluded from the definition. Regardless of CAM definition, there were statistically significant differences in use of CAM by gender, with women reporting higher use than men (*p* < 0.05). For the CAM definition including prayer, there were statistically significant differences in CAM use by race and ethnicity, with Blacks reporting the highest percentage of use (79%) and Asian/Pacific Islanders reporting the lowest percentage of use (57%). When prayer was excluded from the operational definition, racial and ethnic differences were no longer significant. In general, employment status was strongly associated with CAM use, with those employed reporting the highest percentages of use.

Table 2 shows results from the multivariable regression models of CAM use including and excluding prayer. In Model 1 (including prayer), Blacks had significantly higher odds of CAM use than Whites [adjusted odds ratio (AOR) = 2.23, 95% confidence interval (CI) = 1.18, 4.23], after controlling for covariates. CAM use among Hispanic/Latinos and Asians were no different from Whites. Women had significantly higher odds of CAM use than men (AOR = 1.47, 95% CI = 1.02, 2.10), and respondents with incomes of ≥$100,000 had significantly higher odds of CAM use than other lower income groups (AOR = 1.88, 95% CI = 0.96, 3.70). In Model 2 (excluding prayer), gender remained a significant predictor, with women having higher odds of using CAM than men, after controlling for covariates (AOR = 1.59, 95% CI = 1.12, 2.27). Blacks and Hispanic/Latinos were no different than Whites, but Asians had lower use of CAM (AOR = 0.59, 95% CI = 0.36, 0.97). Those in the highest income category had higher odds of use as compared to those in the lowest income category (AOR = 2.27, 95% CI = 1.16, 4.42).

**Table 2:** **Logistic regression results of two operational definitions of complementary and alternative medicine (CAM) use among respondents from the 2014 internet-based Los Angeles County Clinical Services Survey who reported CAM use in the last 12 months, by respondent socio-demographic characteristics (*n* = 1,044)**.

	Model 1: all CAM^a,b^	Model 2: CAM (excluding prayer)^a,c^
	Adjusted odds ratio (95% confidence interval)	Adjusted odds ratio (95% confidence interval)
**Race/ethnicity (*ref: White*)**		
Blacks	2.23 (1.18, 4.23)*	1.33 (0.72, 2.45)
Hispanic/Latino	1.16 (0.76, 1.77)	1.06 (0.70, 1.60)
Asian/Pacific Islander	0.72 (0.43, 1.20)	0.59 (0.36, 0.97)*
**Gender (*ref: men*)**		
Women	1.47 (1.02, 2.10)*	1.59 (1.12, 2.27)**
**Income (*ref: <$15,000–$24,999*)**		
$25,000–$49,999	1.10 (0.58, 2.11)	1.21 (0.65, 2.28)
$50,000–$74,999	1.18 (0.61, 2.27)	1.49 (0.78, 2.83)
$75,000–$99,999	1.33 (0.67, 2.64)	1.72 (0.87, 3.39)
$100,000+	1.88 (0.96, 3.70)*	2.27 (1.16, 4.42)*

*^a^Other covariates included in the weighted model but not presented in this table include age, education employment, income, and health insurance status*.

*^b^Operationalized as all CAM modalities (representing the category “All CAM”), including acupuncture, biofeedback, chiropractic manipulation, cleanse, commercial diet, energy healing/reiki, folk remedies, homeopathy, herbs/botanicals, hypnosis, imagery, lifestyle diet, massage therapy, meditation, megavitamins/dietary supplements, relaxation techniques, self-help groups, self-prayer, spiritual healing by other, yoga, and other (open-ended)*.

*^c^Operationalized as including all CAM modalities in “All CAM” category, except for self-prayer*.

*p < 0.05.

**p < 0.01.

Table 3 lists the top 14 specific CAM modalities used by all respondents and by each of the racial and ethnic groups. The top five CAM types utilized by all respondents were prayer (23%), massage (19%), megavitamins/supplements (14%), cleanse/fast (13%), and yoga (13%). There were significant racial and ethnic differences in use of prayer, megavitamins/dietary supplements, cleanses/fasts, and folk remedies. Blacks reported highest use of prayer (47%), Blacks and Whites highest use of megavitamins/dietary supplements (19% each), Hispanics/Latinos highest use of cleanse/fasts (17%), and Blacks and Hispanic/Latinos highest use of folk remedies (4% each).

**Table 3:** **Top 14 reported complementary and alternative medicine (CAM) types (including prayer) used by respondents, compared by race/ethnicity—data from the 2014 internet-based Los Angeles County Clinical Services Survey, among those who reported CAM use in the last 12 months (*n* = 696)**.

		Black	Hispanic/Latino	White	Asian/Pacific Islander	
CAM type^a,b^	Total (%)	% weighted	% weighted	% weighted	% weighted	*p*-Value^c^
Prayer	22.8	46.9	25.9	16.9	13.6	<0.001
Massage therapy	18.6	26.7	17.9	18.2	15.4	–
Megavitamins/dietary supplements	13.8	19.0	10.0	18.6	12.8	<0.05
Cleanse/fast	12.7	14.3	17.1	9.6	6.1	<0.01
Yoga	12.7	8.1	12.8	13.0	13.8	–
Chiropractic manipulation	13.2	9.7	10.7	17.2	13.3	–
Relaxation techniques	10.2	9.0	11.5	9.7	7.1	–
Lifestyle diets	9.9	9.4	11.5	8.7	7.2	–
Meditation	9.9	11.7	9.1	9.7	10.5	–
Herbs/botanicals	9.0	12.6	7.5	10.9	9.9	–
Acupuncture	7.1	7.6	5.1	8.9	8.3	–
Commercial diet	4.4	4.6	4.8	5.5	1.2	–
Homeopathy	4.7	6.1	3.7	5.4	5.2	–
Folk remedies	2.7	4.0	4.1	0.7	1.7	<0.05

*^a^Number of cases and column percentages do not add up to 100%; only the top 10 CAM combinations are reported among respondents who indicated using one or more CAM modality(-ies)*.

*^b^Respondents were asked, “In the past 12 months which of the following have you used or done? [check all that apply]: acupuncture, biofeedback, chiropractic manipulation, cleanse, commercial diet, energy healing/reiki, folk remedies, homeopathy, herbs/botanicals, hypnosis, imagery, lifestyle diet, massage therapy, meditation, megavitamins/dietary supplements, relaxation techniques, self-help groups, self-prayer, spiritual healing by other, yoga, and other (open-ended)*.*”*

*^c^Weighted chi-squared test*.

Results of a separate sub-analysis (*data not shown in a table*) found that Blacks had a 4.30 higher odds of reporting using prayer as a CAM modality as compared to Whites, after controlling for covariates (AOR = 4.34, 95% CI = 2.34, 7.92; *p*-value < 0.0001). Similarly, after controlling for the same covariates, Hispanics/Latinos had a 1.79 higher odds of using prayer as compared to Whites (AOR = 1.79, 95% CI, 1.07, 2.98; *p*-value < 0.05).

## Discussion

The present study describes CAM use and its patterns in a local urban jurisdiction under two outcome conditions, including and excluding prayer for health. There were four main study findings (based on how CAM was operationalized) that aligned with known patterns of inequalities in health and social services utilization in Los Angeles County.

First, racial/ethnic differences in CAM use emerged when the two operational definitions of CAM were compared. Specifically, when prayer was included in the definition, Blacks had higher levels of CAM use than Whites. But when prayer was excluded, there were no differences between these two groups. Instead, Asians had lower CAM use relative to Whites. These overall findings are generally consistent with and support existing literature about CAM use in diverse populations (11, 23–25). Brown et al. found that including prayer in the definition showed extensive use of CAM among African-Americans (23). Our analysis suggests a similar pattern when prayer was included.

Second, we found significant racial/ethnic differences in the use of specific types of CAM modalities including prayer, megavitamins, cleanse, and folk medicine. This finding makes sense given that many cultures have long histories of folk or traditional medicine, some going back thousands of years, and the use of these culturally rooted modalities or treatments often vary by race/ethnicity. The use of acupuncture, for example, has been found to be higher among Asian Americans (11, 18), whereas the use of *curanderos* are more common among Hispanics/Latinos (11). Racial and ethnic differences in specific types of CAM use have also been observed in other national and California studies (26, 32).

Third, we found other differences in CAM use by gender and socioeconomic status. Women were found to have higher odds of CAM use than men, regardless of how it was operationalized. This finding aligns with a population-based study of California adults that found women using CAM more frequently than men (18). Other surveys using nationally representative data also lend further support to this finding (1, 5, 6, 23, 25, 32, 33). Results from our study also suggest that individuals with incomes exceeding $100,000 have higher odds of reporting CAM use. Education, however, was not independently associated with its use. This latter finding differs from national studies using the NHIS, where education was found to be a strong socioeconomic predictor for using CAM (5, 33, 34). Nonetheless, aligning with our study results, one national study using NHIS data found that higher income is a factor associated with higher CAM use (6). Similarly, a California-based study found that higher income was associated with greater use (albeit only among Whites) (11).

Finally, the present study found that CAM use was relatively higher among all Los Angeles County residents, as compared to national estimates (1–3, 5, 6, 8), regardless of whether prayer was included or excluded in the operational definition of CAM. This discrepancy is not necessarily surprising, given the diversity of Los Angeles County’s population. Prior studies have shown similar high rates of CAM use among California’s diverse populations (11, 18).

### Limitations

The present study was subject to several limitations. First, although the weighted data from this survey produced comparable socio-demographic distributions of Census data, survey respondents were drawn from a proprietary panel of online subscribers/users and thus were subject to selection bias. To mitigate this issue, the Internet panel survey vendor based its sampling and recruitment on target quotas that aligned closely with the U.S. Census demographics for Los Angeles County. Second, the survey was administered via a web-based platform and may have resulted in bias against those who are less technologically savvy. However, socio-demographic quotas were applied to minimize this potential bias. Third, the present analysis relied on a number of self-reported measures; as such, they were subject to recall, reporting, and social desirability bias. Fourth, because specific study goals guided the Internet panel survey, some questions were internally developed rather than adapted from other validated survey items—albeit all questions fielded in the survey were pretested prior to use. Fifth, due to the nature of the survey administration, respondents were not provided with a detailed definition of CAM and related modalities (including prayer/self-prayer). Sixth, the survey separately asked about use of naturopathy. Compared to other CAM modalities which respondents reported as “yes” or “no” to having used them in the last 12 months, naturopathy was reported as a timeframe, which was subsequently recoded as a “yes” or “no” question. This minor difference in variable construction may have led to differences in how respondents answered the question for naturopathy versus other CAM measures. Finally, we did not assess intensity of exposure, how much people value CAM, or rely on CAM. This information could be useful for program planning purposes.

In all, and in spite of these limitations, the present study remains an important, public health surveillance and benchmarking exercise, as the current literature contains little information on CAM use at the local level, especially in a culturally diverse region like Los Angeles County. These findings underscore the importance of using multiple operational definitions of CAM when investigating racial and ethnic differences in use, as well as the need to consider the social and cultural contexts within which this collection of non-mainstream therapies and modalities are used. Another unique contribution of the current study is that we considered a wide variety of modalities (including prayer), some that are not commonly included in other studies (e.g., cleanses/fasts). This attention to a more robust definition of CAM offers opportunities to better benchmark the needs of priority populations, and to inform resource allocation and planning decisions in Los Angeles County.

## Conclusion

The present study sought to understand if including or excluding (self) prayer as part of the operational definition of CAM matters in public health surveillance and benchmarking. We found that including prayer appeared to be associated with CAM use, suggesting that how CAM is defined across studies and in public health practice may have important implications for local health-care delivery decisions and policy development that are based on volume/utilization estimates. Not capturing prayer in surveillance and/or benchmarking activities, for example, can result in under-detection of key reasons why a number of adults failed to seek out proper medical care or inadvertently avoid prevention services (35). Or worse, underestimation of certain CAM modalities can misinform levels to which diverse populations use herb, botanicals, and/or supplements— i.e., types of CAM that may interact with prescribed medications, often leading to serious health consequences and posing particular threat to patients if physicians are unaware of their usage. In the current era of health-care reform, clarity in public health surveillance/benchmarking becomes ever more important, as this activity has implications for both health services planning and assurance of patient safety. Future studies should examine how people value their CAM use and explore the modalities that are associated with delays or substitutions of mainstream medical care. More research is needed to understand which CAM modalities or treatments are congruent with what the medical establishment would recommend.

## Author Contributions

BR, DMU, and TK conceptualized the initial analysis plan. BR supervised the data management, conducted the main analyses using the existing survey data, and led the drafting of the manuscript. TK and DMU contributed to the interpretation and presentation of the data, and provided critical revisions to the content of the manuscript.

## Conflict of Interest Statement

The authors declare that the research was conducted in the absence of any commercial or financial relationships that could be construed as a potential conflict of interest. The reviewer BC and handling editor declared their shared affiliation, and the handling editor states that the process nevertheless met the standards of a fair and objective review.
